# Effect of gender, muscle type and skinfold thickness on myometric parameters in young people

**DOI:** 10.7717/peerj.12367

**Published:** 2021-11-10

**Authors:** Joanna Mencel, Anna Jaskólska, Jaroslaw Marusiak, Katarzyna Kisiel-Sajewicz, Magdalena Siemiatycka, Lukasz Kaminski, Artur Jaskólski

**Affiliations:** Department of Kinesiology, Wroclaw University of Health and Sport Sciences, Poland, Wroclaw, Poland

**Keywords:** Skinfold, Myoton, Viscoelasticity, Methodology, Gender differences, Mechanical properties

## Abstract

**Background:**

The aim of the study was to compare the mechanical properties of three human skeletal muscles: biceps brachii (BB), rectus femoris (RF), and tibialis anterior (TA) at rest measured by myoton device in males (*n* = 16, mean age 21.2 ± 0.6 years) and females (*n* = 16; 21.2 ± 0.9 years) and to investigate the influence of skin and subcutaneous tissue thickness (skinfold thickness, SFT) and gender on myometric parameters of the three skeletal muscles.

**Methods:**

We measured the following mechanical and viscoelastic muscle properties using MyotonPRO^®^: frequency (F [Hz]), decrement (D [log]), stiffness (S [N/m]), relaxation time (R [ms]) and creepability (C [De]). The values of SFT for all selected muscles were assessed by caliper. A mixed-design analysis of variance with gender as between subject comparison was used for assessing the differences between gender and muscles in SFT and each of the myometric parameters separately (F, D, S, R and C). Pearson correlation coefficient or Spearman’s rank correlation coefficient between SFT and myometric parameters was conducted for males, females and males and females together. The level of statistical significance was set at α ≤ 0.05 with Bonferroni correction for multiple comparisons.

**Results:**

The SFT over the RF, TA, and BB muscles in women was statistically significantly larger compared with that of males. In females and males, the SFT over the RF was larger than over the TA and BB, and the SFT over the TA was larger compared with over the BB. The values of F and S recorded for the TA muscle were the highest among the three muscles, while D, C, and R were lowest in TA but highest in the RF muscle in men and women. The values of F and S were smaller in females than in males. Gender comparison of D, C, and R values showed that only D for the RF was significantly lower in females than in males, and C for the RF and TA was significantly larger in females than in males. Some correlation between SFT and myometric parameters were different between males and females. For example, there was a significant, negative correlation between SFT and F for all muscles in females, and a significant, positive correlation between these parameters for BB and TA (not for RF) in males. For pooled data (males and females together), a negative significant correlation between SFT and F was observed for RF and TA (not significant for BB muscle).

**Discussion:**

It is concluded that the TA compared with the BB and RF has significantly greater F and S but the smallest D and C and the shortest R. Gender and muscle differences in the SFT may affect the measurements of muscle properties using MyotonPRO^®^. The relationship between SFT and myometric parameters is different in males and females in the RF, TA, and BB muscles. Therefore, the myometric data should be analyzed in males and females separately.

## Introduction

Many techniques have been successfully implemented to measure the stiffness or elasticity of skeletal muscle. These include ultrasonography (Kubo et al., 2006), shear imaging (Sasaki, Toyama & Ishii, 2014), myometry (Jarocka et al., 2012), shear wave ultrasound elastography (Feng et al., 2018), and the free oscillation technique (Wilson, Wood & Elliott, 1991). Lately, a noninvasive portable device, called MyotonPRO^®^ for measurement of skeletal muscle mechanical properties was introduced (Viir et al., 2006) for which the validity and reliability were already confirmed (Bizzini & Mannion, 2003; Zinder & Padua, 2011). It is used in sport, physiotherapy and medicine (Agyapong-Badua et al., 2016; Gavronski et al., 2007; Lo et al., 2017; Marusiak et al., 2010; Vain & Kums, 2002). The Myoton device applies brief mechanical impulses to the skin above the muscle of interest to include its damped oscillation, from which various parameters can be calculated such muscle tone expressed by frequency (F [Hz]), transverse stiffness (S [N/m]), elasticity expressed by decrement (D [log]), relaxation time (R [ms]) and creepability (C [De]). As these muscles’ properties are measured through the skin, there is a need to know limitations of this method in interpretation of the results. Thus some factors must be considered despite the fact that in such through the skin measurement, each of the factor cannot be separately evaluated quantitively. These factors are muscle architecture, muscle fiber composition as well as properties of the skin and subcutaneous tissue. As all of these factors differ among the muscles and between people of different genders, our study will focus on the comparison of the five myometric parameters in three muscles differing in muscle fiber composition and architecture and in overlying muscle skin and subcutaneous tissue thickness (skinfold thickness; SFT) in young males and females.

### Muscle fiber composition and muscle architecture

Previous studies by other authors have shown that muscle fiber composition and architecture affect its mechanical properties. It has been documented (Cui, Perreault & Sandercoock, 2007; Walmsley & Proske, 1981) that the slow twitch (ST) and fast twitch (FT) fibers differ in their stiffness with greater short-range stiffness (just after an imposed length change) observed in the ST fibers. However, the short-range stiffness has little effect on transverse muscle stiffness (as measured by Myoton), while the collagen around individual muscle fibers may have it (which may increase muscle stiffness. Kovanen, Suominen & Heikkinen (1984) showed that the soleus muscle and ST area of the gastrocnemius muscle are more collagenous than the rectus femoris (RF) and the FT area of the gastrocnemius muscle, at the level of both the perimysium and endomysium in rats. Thus, it can be expected that muscles with different ST and FT fiber content (such as biceps brachii (BB), RF, and tibialis anterior (TA); Johnson et al., 1973a; Johnson et al., 1973b) will differ in mechanical characteristics.

In terms of muscle architecture as a factor affecting the measurement of muscle mechanical properties, it should be noticed that the parallel-fusiform muscle (such as the BB) has longer muscle fibers and fewer fibers per anatomical cross-sectional area (and thus less collagenous fibers examined at dedicated measurement points) than the pinnate muscle (such as RF and TA), and these muscles differ in anatomical and physiological cross-sectional area (Fidelus, Dukaczewska & Jaskólska, 1978; Fukunaga et al., 1992). Indeed, it has been shown that the stiffness of the human BB muscle is lower than the stiffness of TAand RF muscles (Agyapong-Badua et al., 2016; Gavronski et al., 2007) but among the three muscles, differences in the other myometric parameters are not known.

### Skin and subcutaneous tissue thickness and measurement of muscle mechanical properties

As previously reported (Cooper et al., 2014; Fröhlich-Zwahlen et al., 2014; Jaskólska et al., 2004), muscle skinfolds can affect the human muscles’ mechanical properties outcomes measured with various devices from the skin surface. A negative correlation was found between the thickness of subcutaneous adipose tissue and RF hardness (Chino & Takahashi, 2016), but no relationship was observed between subcutaneous fat tissue and muscle tone as measured by tonometer (Ylinen et al., 2006) and Myoton (Gapeyeva et al., 2000). Agyapong-Badua et al. (2016) found that the correlation between Myoton parameters of the RF and fat thickness ranged from *r* = −0.01 to −0.49 within the four groups: young females, old females, old males, and young males. Fröhlich-Zwahlen et al. (2014) showed a generally negative correlation between subcutaneous thickness and stiffness in RF, vastus lateralis and biceps femoris (BF) muscle, between subcutaneous thickness and tone in RF and BF as well as between subcutaneous thickness and elasticity of BF. There were no correlation between those parameters in TA muscle. The results presented above are confusing and appear to vary across measurement tools, muscles, and groups of participants.

### Gender-related differences in skin and subcutaneous tissue properties and thickness, in muscle fiber composition and muscle mechanical properties

It has been shown that the male’s skin is thicker and stiffer than that of females (Shuster, 1972; Shuster, Black & McVitie, 1975). Study of Firooz et al. (2012) have presented that skin elasticity is higher in females than in males. The subcutaneous tissue thickness (SFT) is generally higher in females than in males (Tur, 1997). Tchoukalova et al. (2010) have found that women store more fat in subcutaneous areas especially in the gluteal and femoral depots. With regard to muscle fiber composition, it has been found that muscles in females contain generally more ST muscle fibers being more collagenous (Kovanen, Suominen & Heikkinen, 1984) then FT, while males have prevalence of FT fibers in their muscles (Haizlip, Harrison & Leinw, 2015). Some differences were also found in muscle properties between males and females (Miller et al., 1993; Sale et al., 1987). Gender differences in muscle mechanical properties measured using different laboratory-based technologies are conflicting in the literature. For example, sheer wave elastography demonstrated greater stiffness in females (Eby et al., 2015; Chen et al., 2017), while the opposite was found (greater stiffness in males than in females) in VL using Myoton-3 (Wang et al., 2015; Fröhlich-Zwahlen et al., 2014) and in RF, TA, BF, and GM (Fröhlich-Zwahlen et al., 2014; Agyapong-Badua et al., 2016; Zinder & Padua, 2011), and the structural stiffness in triceps surae muscle (Blackburn et al., 2006). In contrast, Agyapong-Badua et al. (2016) did not find gender differences in the BB muscle. Moreover, Fröhlich-Zwahlen et al. (2014) and Agyapong-Badua et al. (2016) found that men had greater muscle tone compared with women, while women displayed greater elasticity than men in RF muscle. Results of Blackburn et al. (2006) of a greater structural stiffness and material modulus in males than females suggests that these differences are likely attributable to gender differences in tendon stiffness and muscle structure. The disagreements among the studies on gender influence as well as the differences in the effect of SFT and skin properties may be due to the methods used and muscles studied. Given this, and in light of the growing number of articles on the usefulness of myometric measurements, there is a need to explore the potential limitations of this method, especially the sensitivity to gender and skinfold thickness. These are important issues in the context of comparing the results of different studies that do not pay attention to the above.

### Aim of the study

The aim of the study was to compare the mechanical properties of three human skeletal muscles (BB, RF, and TA) at rest measured by the MyotonPRO^®^ in young males and females and to investigate the influence of SFT and gender on myometric parameters of the three skeletal muscles.

Among the muscle differences in Myoton parameters considered, we assumed that TA muscle (having almost double the ST fiber content as compared with RF and BB) with pinnate muscle architecture would have the largest values of frequency/muscle tone (F) and muscle stiffness (S) but the lowest values of decrement (D), creepability (C), and relaxation (R).

In terms of gender and muscle differences in ST fiber content (characterized by greater stiffness than FT fibers) and larger skin thickness and stiffness in males than females, we assumed that there would be a different relation between SFT and myometric parameters in the three muscles between males and females.

## Materials & Methods

### Subjects

Thirty-two untrained, healthy volunteers (16 men and 16 women) were tested in this study. The values of anthropometric parameters of the participants are described in Table 1.

**Table 1:** Anthropometric characteristics of the subjects.

Gender	N	Age [years]	Body weight [kg]	Body height [m]	BMI [kg/m^2^]
Female	16	21.2 ± 0.9	57 ± 5.6	1.66 ± 0.07	23.6 ± 1.8
Male	16	21.2 ± 0.6	77.1 ± 9.8	1.80 ± 0.07	23.6 ± 2.11

The inclusion criteria were age between 18 to 29 years, self-reported lack of vigorous or professional physical activity in the past 2 years, no history of surgery and serious injuries to the upper and lower limbs. Exclusion criteria were a state of injury, chronic disease requiring medication, skin conditions in measurement areas, and body mass index (BMI) ≥30 (the last factor, according to the recommendations of Gapeyeva & Vain (2008). All participants were well informed of the study’s aim and procedure and provided written consent prior to the experiment. The study was approved by the Ethical Committee of the University School of Physical Education in Wroclaw (reference number of project: 29/2013) and was conducted in accordance with the Declaration of Helsinki.

### Experimental procedures

The participants were placed in the supine position, lying with their upper limb extended along the trunk and in supination and the lower limb extended and in the intermediate position. Soft towels were used to help participants lie in this position and relax during the experiment.

When the participant was positioned correctly, the points above the selected muscles bilaterally were identified (first for the right side and then for the left side). For the BB muscle (short head) and TA muscle, the measurement point was chosen in the central part of the muscle belly (Gapeyeva & Vain, 2008) and for RF at two-thirds distally between the anterior superior iliac spine and the superior pole of the patella (Agyapong-Badua et al., 2016). Before the points were marked by a skin-safe pen, the investigator used palpation to check that the place was correctly chosen above the muscle of interest. After the points were marked, the SFTs of these points were measured for all selected muscles using a caliper first for the right side, then for the left side in order: skinfold over BB, RF and TA (to the nearest 0.2 mm and with a constant pressure of 10 gms/sq. mm; Tanner/Whitehouse Skinfold Caliper). Skinfold testing involved taking a caliper, pinching the skin with subcutaneous fat, pulling the skinfold away from the underlying muscle, and measuring the thickness of the skinfold with the caliper (Torun & Mutluay, 2017).The next step involved myometric measurement and the participant received oral instruction to relax. The testing end of the MyotonPRO^®^ device (Myoton AS, Estonia) was placed perpendicular to the skin surface above the marked point, and the device was lowered into the measurement position and held steadily. During this time, the device automatically performed a series of 10 mechanical taps (for more details, see (Marusiak et al., 2018), from which the mean value of the muscle response was used to calculate the five myometric parameters. These parameters consisted of frequency (F [Hz]), characterizing nonneural tone; decrement (D [log]), characterizing elasticity (as the ability of a muscle to recover its shape after being deformed—the lower the value, the higher elasticity and less damping of the tissue’s oscillation), and transverse stiffness (S [N/m]), indicating the ability of the muscle to resist an external force that modifies its shape. Two other parameters (which are rarely documented) characterize the tissue’s viscoelastic properties. They are relaxation time of mechanical stress (R [ms]), as the time needed for a reference amount of deformation to occur under a suddenly applied reference load, and the ratio of relaxation time to deformation time, characterizing creepability (C [De]). In the more fluid-like materials, less time is required for flow (shorter relaxation time), giving a lower Deborah number.

Then again, the investigator (blinded to the results of earlier measurements) measured the skinfolds at the previously marked points in the same way as the first time. The mean value of the two measurements was used for further analysis. All of the above measurements were performed by the same, experienced investigator.

### Statistical analysis

Data were analyzed using SPSS 22.0 software (IBM, Armonk, NY, USA). The Shapiro–Wilk test was performed to estimate the distribution of values. A mixed-design analysis of variance (ANOVA) with gender (level 2, males and females) as a between-subject comparison and muscles (level 3; BB, RF and TA) and body side (level 2, right and left) as within-subject comparison was used. The analysis did not show a significant effect of body side on any of the analyzed parameters, so further tests were conducted on a sample size of 64 cases (without further consideration of sides). Thus for assessing the differences between gender and muscles in skinfold thickness and each of the myoton parameters separately (F, D, S, R and C), a mixed-design ANOVA for pooled data (*n* = 64 cases) was apply (gender as a between-subject comparison while muscles as within-subjects). ANOVAs were also used separately for male and female to determine the effect of the muscle factor on all the measured parameters. The Greenhouse-Geisser correction was applied where sphericity was violated and follow-up comparisons were conducted using Bonferroni-adjusted multiple comparisons. The partial eta-squared (${\eta }_{P}^{2}$) was used as a measure of effect size.

Pearson correlation coefficient or Spearman’s rank correlation coefficient between SFT and parameters of myometry was conducted for males, females and males and females together (*n* = 64). The level of statistical significance was set at α ≤ 0.05. Data are presented as means and standard deviations. The achieved power of the results of the pairwise comparisons is 0.88 and was calculated in G*Power tool (Faul et al., 2007).

## Results

### SFT over three muscles in males and females

Results of ANOVA have shown a significant effect of muscle for SFT F(1.46, 90.90) = 378.19, *p* < 0.001, ${\eta }_{P}^{2}=0.859$. There were also significant effect of gender –F(1, 62) = 176.644, *p* < 0.001, ${\eta }_{P}^{2}$ = 0.740 and significant effect of interaction between gender and muscle –F(2, 62) = 35.269, *p* < 0.001, ${\eta }_{P}^{2}$ = 0.363 for SFT.

Similarly, a significant effect of muscle for SFT were shown in ANOVAs performed separately for female and male (F(1.198, 19.175) = 95.849, *p* < 0.001, ${\eta }_{P}^{2}$ = 0.857; F(2, 32) = 57.662

*p* < 0.001, ${\eta }_{P}^{2}$ = 0.783 respectively).Further analysis has shown that SFT over RF, TA, and BB muscles in females was significantly larger compared with males (*p* ≤ 0.05; Table 2).

**Table 2:** Mean values with standard deviation (M (SD)) of skinfolds thickness and myometric parameters for rectus femoris (RF), tibialis anterior (TA), and biceps brachii (BB) muscle in females (F, *n* = 32) and males (M, *n* = 32) and results of pair comparison between females and males and between muscles. Bolded values are statistically significant.

Parameter	Gender	RF	TA	BB	*P* value					
					Females *vs.* males			RF *vs.* TA	RF *vs.* BB	TA *vs.* BB
					RF	TA	BB			
SFT	F	18.7 (5.00)	10.1 (2.11)	6.8 (1.69)	**<0.001**	**<0.001**	**<0.001**	**<0.001**	**<0.001**	**<0.001**
	M	10.4 (3.40)	6.57 (2.05)	4.14 (1.64)				**<0.001**	**<0.001**	**<0.001**
F [Hz]	F	13.7 (0.78)	18.6 (1.50)	14.1 (0.84)	**<0.001**	**=0.001**	=0.743	**<0.001**	=0.207	**<0.001**
	M	15.1 (0.91)	19.9 (1.63)	14.0 (0.66)				**<0.001**	**<0.001**	**<0.001**
S [N/m]	F	245 (20.9)	369 (39.3)	227 (19.2)	**<0.001**	**=0.001**	=0.235	**<0.001**	**<0.001**	**<0.001**
	M	282 (27.7)	418 (57.8)	221 (21.8)				**<0.001**	**<0.001**	**<0.001**
D [log]	F	1.13 (0.21)	0.81 (0.08)	1.08 (0.12)	**<0.001**	=0.51	=0.497	**<0.001**	=0.02	**<0.001**
	1M	11.42 (0.22)	10.84 (0.09)	1.09 (0.13)				**<0.001**	**<0.001**	**<0.001**
C [Db]	F	1.30 (0.09)	0.93 (0.09)	1.20 (0.07)	**=0.006**	**=0.04**	=0.298	**<0.001**	**<0.001**	**<0.001**
	M	1.23 (0.08)	0.84 (0.12)	1.18 (0.05)				**<0.001**	**=0.004**	**<0.001**
R [ms]	F	21.7 (1.53)	14.9 (1.58)	20.9 (1.43)	**<0.001**	=0.94	=0.846	**<0.001**	**=0.002**	**<0.001**
	M	20.02 (1.60)	14.3 (3.15)	20.9 (1.12)				**<0.001**	**=0.007**	**<0.001**

**Notes.**

SFT skinfold thickness F frequency S stiffness D decrement C creepability R relaxation time

The same differences between SFTs for different muscles were noted separately in the male and female group. The SFT over RF was larger than over TA and BB (*p* ≤ 0.05), and the SFT over TA was larger compared with BB (*p* ≤ 0.05; Table 2).

### Comparison of myometric parameters among muscles in males and females

Results of ANOVA have shown that there are differences between muscles for each of the myometric parameters (Frequency F(1.45, 90.27) = 689.37, *p* < 0.001, ${\eta }_{P}^{2}$ = 0,917; Stiffness F(1.38, 85.56) = 607.59, *p* < 001, ${\eta }_{P}^{2}$ = 0,907; Decrement F(1.58, 98.11) = 130.45, *p* < 0.001, ${\eta }_{P}^{2}$ = 0,678; Relaxation time F(1.46, 90.68) = 314.86, *p* < 0.001, ${\eta }_{P}^{2}$ = 0,835 and Creepability F(1.71, 106.40) = 424.93, *p* < 0.001, ${\eta }_{P}^{2}$ = 0,873). Similar results were noted for ANOVAs calculated separately for females and males. In the female group, the results were as follows: Frequency F(1.441, 44.661) = 278.475, *p* < 0.001, ${\eta }_{P}^{2}$ = 0.900; Decrement F(1.393, 43.195) = 41.308, *p* < 0.001, ${\eta }_{P}^{2}$ = 0.571; Stiffness F(1.456, 45.136) = 334.606, *p* < 0.001, ${\eta }_{P}^{2}$ = 0.915; Creepability F(1.630, 50.523) = 190.299, *p* < 0.001, ${\eta }_{P}^{2}$ = 0.860; Relaxation time F(2, 62) = 258.632, *p* < 0.001, ${\eta }_{P}^{2}$ = 0.893. The results of ANOVA for males were as follows: Frequency F(1.372, 35.678) = 329.399, *p* < 0.001, ${\eta }_{P}^{2}$ = 0.927; Decrement F(1.640, 42.629) = 83.188, *p* < 0.001, ${\eta }_{P}^{2}$ = 0.762; Stiffness F(1.238, 32.200) = 224.005, *p* < 0.001, ${\eta }_{P}^{2}$ = 0.896; Creepability F(2, 52) = 198.007, *p* < 0.001, ${\eta }_{P}^{2}$ = 0.884; Relaxation time F(1.287, 33.469) = 88.137, *p* < 0.001, ${\eta }_{P}^{2}$ = 0.772.

Further comparisons have shown that the values of frequency (F) and muscle stiffness (S) recorded for the TA muscle were the highest among the three muscles (*p* ≤ 0.05; with the lowest values for the BB except F for RF in females; *p* ≤ 0.05). Decrement (D), creepability (C), and relaxation (R) were the lowest in TA but the highest in RF muscles in males and females (*p* ≤ 0.05; Table 2). The values of F and D in females for the BB muscle were not significantly different from those recorded for the RF muscle (*p* > 0.05; Table 2).

The values of F and S were smaller in females than in males, with a statistically significant difference for RF and TA muscles (*p* ≤ 0.05) but not for BB (*p* > 0.05; Table 2).

Gender comparison of the values of D, C, and R showed that only D for the RF was significantly lower in females than in males, and C for the RF and TA was significantly larger in females than in males (*p* ≤ 0.05; Table 2). There were no statistically significant differences between males and females in the BB muscle’s Myoton parameters (*p* > 0.05; Table 2).

### Relationship between SFT and myometric parameters in RF, TA and BB muscles in males and females

After the SFT over RF and TA muscle in male group and SFT over BB and TA for pooled data did not meet the criteria for normality of distribution, the relationships for them with myometric parameters were determined by Spearman’s correlation analysis. Nonparametric correlation analysis was also performed between SFT over RF and two myometric parameters in the female group: C and R (the distribution of values for these parameters did not meet normality criteria). Pearson correlation analysis was performed for the remaining parameters. Note that the type of correlation analyses is indicated in the Table 3. Frequency was negatively related to SFT in RF, TA, and BB muscles in females (all correlation coefficients were statistically significant; *p* ≤ 0.05), while in males, the respective coefficients were positive and statistically significant in the TA and BB muscles (*p* ≤ 0.05) but not in RF (*p* > 0.05; Table 3).

**Table 3:** Results of correlations analysis between skinfold thickness and myometric parameters for three tested muscles for females (F), males (M) and females and males data together (All). Bolded numbers are statistically significant.

Parameter	Gender	RF			TA			BB		
Frequency (F) [Hz]	F	**–**	**0.534**		**–**	**0.340**		**–**	**0.435**	
	M		0.150	^#^		**0.465**	^#^		**0.563**	
	All	**–**	**0.505**		**–**	**0.414**	^#^		0.180	^#^
Stiffness (S) [N/m]	F	–	0.210		–	0.236		–	0.085	
	M	–	0.054	^#^		**0.628**	^#^		**0.620**	
	All	**–**	**0.460**		–	0.201	^#^		**0.335**	^#^
Decrement (D) [log]	F		**0.337**			**0.743**			**0.438**	
	M	**–**	**0.525**	^#^		0.176	^#^		**0.340**	
	All	**–**	**0.447**			**0.463**	^#^		0.224	^#^
Creepability (C) [Db]	F		**0.650**	^#^		0.329			**0.426**	
	M	–	0.201	^#^	**–**	**0.502**	^#^	**–**	**0.392**	
	All		**0.363**			0.186	^#^		0.050	^#^
Relaxation (R) [ms]	F		**0.608**	^#^	-	0.278			0.295	
	M	–	0.072	^#^	**–**	**0.361**	^#^	**–**	**0.601**	
	All		**0.426**			0.061	^#^	–	0.201	^#^

**Notes.**

RF rectus femoris TA tibialis anterior BB biceps brachii

# Spearman’s rho was calculated; the lack of ‘#’ means that Pearson correlation was calculated.

When pooling the data of males and females, the SFT correlated negatively with F for the RF and TA muscles, but for the BB muscle, the correlation was not significant.

Similarly, negative correlations were found between stiffness and SFT in females (*p* > 0.05) but was positive in males; however, only statistically significant correlation coefficients were recorded in males in the TA and BB muscles (*p* ≤ 0.05; Table 3). For all participants together (*n* = 64), the relationship between SFT and S for the RF muscle was negative, as in females and males, but in contrast and statistically significant, for the TA was similar to the value recorded for females (opposite to that found in males), while in the BB muscle was statistically significant and positive as in males (but with a much smaller value; Table 3).

Decrement was positively related to SFT in males and females (except for the RF muscle in males, which was negative and statistically significant; *p* ≤ 0.05). The correlation coefficients in RF, TA, and BB muscles for females were statistically significant (*p* ≤ 0.05), while in males, a positive and statistically significant correlation was found in the BB muscle (*p* ≤ 0.05; Table 3). The correlation coefficients for all participants in TA and BB muscles were positive as separately calculated for females and males but not statistically significant in the BB muscle. For the RF, the correlation coefficient was similar to that found in males (Table 3).

Correlation coefficients between the creepability parameter and SFT in the three muscles in females and males were inverse to those recorded for the frequency parameter. This means that the R values were positive in females (all statistically significant; *p* ≤ 0.05) but negative for males (*p* ≤ 0.05), with a non-statistically significant correlation for the RF muscle (*p* > 0.05; Table 3). When correlation coefficients were calculated for all pooled data (men and women together), the coefficients were positive (as found in females) but statistically significant only for the RF (Table 3).

For the relaxation parameter, a statistically significant correlation with SFT was recorded in the RF muscle in females (positive correlation) and in the BB and TA muscles in males, which was opposite to the correlation coefficient in females (Table 3). The other correlations in males and females were not statistically significant (Table 3). For pooled data (women and men together; *n* = 64), there was only one statistically significant and positive correlation coefficient in the RF muscle (as found in females; Table 3).

## Discussion

In agreement with our assumptions, we found that (i) frequency and stiffness were greatest in the TA muscle, while decrement, relaxation, and creepability were the lowest in TA compared with RF and BB muscles and that (ii) the impact of SFT on the measured parameters is different not only among BB, RF, and TA muscles but also between males and females, indicating the importance of separate assessment and data analysis between males and females on the influence of SFT on muscle mechanical properties.

### Comparison of mechanical properties between muscles

Our results of the mechanical and viscoelastic properties of the three muscles are in agreement with our assumptions. The values for tone, stiffness, and elasticity (decrement) reported in the present study for RF and TA muscles were in a range comparable with those found by Fröhlich-Zwahlen et al. (2014) in a group of males and females pooled together (age 53 ± 10 years). Similar to our findings, the values of the stiffness and frequency reported by the authors were larger in the TA than in the RF, while decrement was smaller in the TA than in the RF. The results of the BB muscle in the present study also agree with those reported in the literature by Agyapong-Badua et al. (2016) and Gavronski et al. (2007). This means that the stiffness of the human BB muscle is lower than in the TA and RF muscles. Our results for creepability and relaxation characterized viscoelastic properties in the three muscles cannot be compared with the findings of other studies, because to our best knowledge, such data have not yet been reported in the literature. As we hypothesized, we found the smallest values of creepability and relaxation for the TA muscle and largest for the RF muscle. The reason for this high frequency and transverse stiffness and small decrement, creepability, and relaxation for TA is probably complex but could be related to the muscle’s location and smaller anatomical cross-section area as compared with RF (Fidelus, Dukaczewska & Jaskólska, 1978). It could also be related to its pinnate architecture and high proportion of type I fibers (ST) that are stiffer than those in type II (FT) as they are more collagenous (Kovanen, Suominen & Heikkinen, 1984). These studies have indicated the important role of collagen and the intramuscular structure of connective tissue, including endomysial collagen in relation to skeletal muscle function (Kovanen, Suominen & Heikkinen, 1984). It appears that the amount, type, and structure of intramuscular collagen may explain such differences in the elastic properties of fast tetanic muscles compared to slow tonic muscles as the higher compliance (inverse of stiffness) of fast muscles. In addition, SFT over the TA muscle is also rather small. On the other hand, the smaller absolute differences in mechanical properties between the RF and BB muscles can be related to muscle architecture (RF is a pinnate while BB a parallel oblique), the proportion of ST to FT type (30% ST in RF compared with 40–50% in BB; Johnson et al., 1973a; Johnson et al., 1973b) and thickness of SFT (RF is covered by a SFT that is almost twice as large as that above the BB muscle).

The mechanical parameters measured by Myoton indicate that among the three muscles tested, the TA muscle has the largest intrinsic tension at the cellular level (highest frequency), which partially results from its shortest relaxation and smallest creepability. The TA is also the most stiff (least compliant) with the greatest elasticity (smallest decrement value). There are much smaller or no differences in mechanical properties between the RF and BB muscles measured *in vivo* through the skin.

### Differences in mechanical properties of skeletal muscle between females and males

In terms of gender-related differences between the mechanical properties of muscles, we found that in females, the frequency (intrinsic tension) and decrement did not differ significantly between the RF and BB muscles (although they did in males). *C* reepability did not differ between the RF and BB muscles in males, although it did in females. The values of frequency, stiffness, and decrement were lower and creepability and relaxation larger in females than in males in both the RF and TA muscles (there was no significant difference in TA in decrement or in relaxation in both muscles). The principal outcome interpretation criterion is as follows: the higher the values of F and S, the greater the tension and stiffness of the examined soft-tissue structure. The lower the D value, the smaller the dissipation of mechanical energy during oscillation and the higher the elasticity of the muscle (Gavronski et al., 2007), tendon, or fascia. The lower the relaxation value, the higher the tension. Thus, the results have shown that the parameters of myometry are significantly different between young healthy women and men for RF and TA muscles at rest, and men had higher nonneural tone and transverse stiffness in those muscles compared with women. The above results indicate that the thicker and larger ST fiber content in female as compared with male muscles (Carter et al., 2001; Haizlip, Harrison & Leinw, 2015; Nygaard, 1981; Roepstorff et al., 2006; Wüst et al., 2008) has a smaller impact on gender-related differences as measured through skin mechanical properties than other factors, such as skinfold and skin properties and thickness. This is to some extent confirmed by the lack of gender differences in the mechanical properties of the BB muscle, which contains roughly 50% of ST and FT muscle fibers and is covered by the smallest (compared with RF and TA) SFT in both females and males. In addition, as skin elasticity has been shown to be greater in females than in males (Firooz et al., 2012), there is greater elasticity (lower decrement) in females than in males, especially in the RF muscle (which has the fewest ST fibers and is covered by the largest SFT compared with the TA and BB muscles).

Our data on RF are in agreement with the study by Wang et al. (2015) which showed greater muscle transverse stiffness in VL - different head of the quadriceps femoris muscle, in men compared with women. The authors concluded that the greater muscle stiffness in men could be related to their higher physiological cross-sectional area and greater muscle mass compared with women.

The values of all myometry parameters for BB in men were similar to those reported by Mooney, Warner & Stokes (2013) in their study on 21 men, aged 25.8 ± 4.1 years. The results for RF were similar to those reported by Mullix, Warner & Stokes (2012) for 21 healthy males, which also indirectly indicates the repeatability of the results obtained by the myoton device. Our results are similar to those presented by Agyapong-Badua et al. (2016), who showed no significant difference in elasticity, tone, or stiffness between young women and men for the BB muscle and differences for all of those parameters for the RF muscle.

### Skinfolds and the mechanical properties of skeletal muscle

We found a different relationships between SFT and myometric parameters among the three muscles in males and females. The reason for the discrepancy in the muscles’ mechanical properties between males and females could lie in differences in superficial tissue, body composition, and skin properties. In the present study, the SFT was larger over the RF than over the TA and BB muscles, the SFT of the TA was larger than that over the BB, and the skinfolds were thicker in women than in men, which is in agreement with data provided by Durnin & Womersley (1974). Women store more fat in subcutaneous areas and this could potentially influence the results of the *in vivo* assessment of mechanical properties through the skin. Moreover, a study by Fröhlich-Zwahlen et al. (2014) showed a significant negative correlation between subcutaneous thickness and the frequency and transverse stiffness of the RF muscle, as assessed by myometry, in group of 40 subjects (20 poststroke patients and 20 matched controls, aged 52.5 ± 10.5 years, 11 men and 9 women in each group). This relationship was also identified for the VL muscle (men had a higher transverse stiffness and lower value of subcutaneous thickness than women did). However, the authors pooled the data from different age groups and genders. Thus, it is impossible to draw a valuable conclusion on the influence of skinfold on the measurement of muscle mechanical properties in males and females. This is because the subcutaneous fat thickness, skin thickness and stiffness varies with gender and age. Thus, a pooled correlation analysis would change the relationship between the muscle parameters and fat, which is evident in our study.

With the exception of stiffness in the RF muscle and decrement in the BB muscle, the correlation coefficients between SFT and parameters of mechanical and viscoelastic properties in females were opposite or in the reverse direction to that reported in males. In females, the larger the SFT, the lower frequency/muscle tone (and stiffness without statistical significance), while in males, the larger the SFT, the greater the intrinsic muscle tone and stiffness. For decrement, creepability, and relaxation, the correlation coefficients are in the reverse/opposite direction to that of frequency and stiffness in females and in males. Although some authors did not find gender-linked differences in skin properties (Ezure et al., 2011; Ishikawa, Ishikawa & Miyachi, 1995) and epidermal thickness (Gambichler et al., 2006; Mogensen et al., 2008), Sandby-Møller, Poulsen & Wulf (2003) recorded a thicker cellular epidermis in males than in females. In addition, Luebberding, Krueger & Kerscher (2014) found that the mechanical properties change differently in males and females over their lifetime and that the skin of females is less distensible but has a greater ability to recover after stretching in comparison with male skin.. This means that skin elasticity in females is greater than in males (Firooz et al., 2012). These results found some confirmation in a smaller decrement female RF muscle in our study. Moreover, the results of Shuster and coauthors (Shuster, 1972; Shuster, Black & McVitie, 1975) showed that showed that the skin in males has more collagen fibers than in females at all ages, and the skin collagen is less densely packed in females than in males, supposedly due to androgen, as skin collagen density is increased in patients with primary cutaneous virilism (Shuster, 1972). The findings of Shuster and coauthors (1975) found that that collagen is a major component of skin thickness, and there is a relationship between skin collagen and dermal thickness in males of all ages but not in females younger than 60 years. The authors found that male skin is about 20% thicker and thus stiffer than female skin. Indeed, in the TA and BB muscles (over which there is thin SFT) for males, the thicker the skinfold (and larger the collagen content), the greater the frequency (muscle tone) and stiffness. When skinfold is thin, as in the case of the TA and BB muscles in males, there is a greater contribution and effect of skin collagen fiber properties on the measurement of muscle mechanical properties, because the collagen fibers are more densely packed in males and have greater stiffness compared with subcutaneous fat tissue and relaxed muscle fibers (as was the case in the present study). It was observed for RF, over which the skinfold is twice as large as that in the BB and TA where this effect was not observed (except for decrement). In contrast, in females there is thicker subcutaneous fat tissue, less densely packed collagen fibers in the skin, and no relationship between collagen content and skin thickness, which is inverse to the relationship observed in males between the SFT and myometric parameters, especially in the RF (which is covered by a thicker skinfold). This is why in the RF, the correlation coefficients were statistically significant (except for stiffness) in females but not in males (except for decrement). In contrast to the RF muscle, in the TA, three correlation coefficients were not statistically significant (stiffness, creepability, and relaxation) in females, while for males, only one was not significant (decrement). In the BB muscle in females, there were no statistically significant correlations for stiffness and relaxation, while in males, all of these were statistically significant (keeping in mind that with a couple of exceptions, the sign of the correlations in males was different than in females, *i.e.,* the opposite direction). Although most of the correlations were statistically significant, they were weak (low) to strong (high) (0.329–0.743). The square of the correlation coefficient is typically expressed in percentage terms and called a coefficient of determination. For example, TA skinfold and decrement in females was 0.743, and hence the *R*^2^ value would be 0.55. The interpretation of this number is that 55% of the variation in the decrement (the dependent variable in this case) is related to or can be explained by the variation of the skinfolds (the independent variable). Our data are similar to those published by Agyapong-Badua et al. (2016) for tone of RF (*r* =  − 0.48). On the other hand, Agyapong-Badua et al. [9] did not report any correlations between fat thickness and mechanical parameters of BB and RF. The differences might be seen in different fat-thickness measurements. They measured only RF fat thickness using ultrasound imaging, whereas in our study, the caliper was used. When the data from our study are compared with those of Fröhlich-Zwahlen et al. (2014), the same tendency for correlation between SFT in females for stiffness and tone in RF is observed.

Based of the indicated differences in the relationship between SFT and myometric parameters of three muscles in females and males, it should be pointed out that myometric data for women and men should not be combined and considered together. Analyzing combined data from males and females may lead to false conclusions, as indicated by the dispersion/scattering of the correlation coefficients calculated in our study for the entire group of participants (males and females together) compared with those calculated for females and males separately (*e.g.*, when coefficients are in opposite directions in males and females). Similarly, if a result is qualitative in males and females (*e.g.*, different SFT and mechanical properties of skeletal muscle in males *vs.* females), analyzing data in aggregate may lead to erroneous conclusions for both genders. Furthermore, analyzing results in one gender but generalizing results to both males and females may also result in erroneous conclusions, which has been shown in our study.

### Limitations and implications of our findings

Our study has some limitations that should be identified. The state of muscle relaxation was not measured objectively (*e.g.*, using electromyography), so it was not possible to be certain that the muscles during myometric measurements were in an ideal resting state. Females were not asked in what phase of the menstrual cycle they were in during the myometric measurements, and it is known that hormone levels might influence skeletal muscle stiffness (Bell et al., 2012). Another limitation of our study, may appear to be the use of a skinfold caliper to determine size of subcutaneous body fat instead of ultrasound imaging or computed-tomography measurements.

However, according to the study of Orphanidou et al. (1994), this seems reasonable. Studies by these authors indicated that relative agreement in the measurement of subcutaneous body fat between caliper and computed tomography measurements was better to that between ultrasound and computed tomography measurements (Orphanidou et al., 1994). Their finding enhances the potential use of skinfold calipers especially in participants or patients who are not overweight when performed by an experienced investigator, as was the case in our study.Our results have shown that researchers and clinicians should pay attention to SFT in relation to myometric measurements. Some of the myometric parameters of the skeletal muscle were related to the thickness of skinfolds, and this is clinically relevant, as this factor is not taken into consideration when mechanical parameters of skeletal muscle are evaluated in clinical routine. This is the strength of our research, especially in light of the increasingly used, easy-to-handle myoton device. Potentially, it could be even more important in different groups of participants (*e.g.*, those with a larger BMI, older people with different properties of SFT or individuals with disease), but this needs further research. In addition, our study is in alignment with the guidelines of the National Institutes of Health to include both gender in preclinical research (Miller et al., 2017); taking into account our findings of the gender-related influence of SFT on the assessment of muscle mechanical properties, the evaluation of myometric muscle characteristics must be done separately for males and females, at least for some muscles.

Because of relatively small group of participants, our study should be define as non-conclusive preliminary type and our results are applicable to healthy young adults.

## Conclusions

The tibialis anterior muscle compared with the biceps brachii and rectus femoris has significantly greater muscle tone and stiffness but the smallest decrement (biggest elasticity) and creepability and the shortest relaxation.

Gender and muscle differences in the skin and subcutaneous tissue thickness may affect the measurements of muscle properties using Myoton.

The relationship between skinfold thickness and myometric parameters is different between males and females in the rectus femoris, tibialis anterior, and biceps brachii muscles.

## Supplemental Information

10.7717/peerj.12367/supp-1 Supplemental Information 1 Raw data Myometric data and skinfold values measured over the biceps brachii (BB), rectur femoris (FR) and tibialis anterior (TA) muscles of the subjects.

## Additional Information and Declarations

### Data Availability

The following information was supplied regarding data availability: The raw data is available in the Supplemental File.
